# Supplementary material for: Spinal circuit mechanisms constrain therapeutic windows for ALS intervention: A computational modeling study
Source: Neurobiol Dis. 2026 Feb;219:107253. doi: 10.1016/j.nbd.2025.107253 (PMC12858429; doi:10.1016/j.nbd.2025.107253)
Supplement: MMC S1 — The supplementary material contains network and neuronal characteristics for reproducibility, and additional statistics. [file mmc1.pdf]

Supplementary Material

Appendix A. Network and Neuronal Characteristics

Table A.1: Characteristics per neuron population.

**Population**	**# of neurons**	**Firing behavior**	**Drive (pA)**
RG Flx exc	240	Bursting	450 ± 112
RG Flx exc	560	Tonic	720 ± 180
RG Flx inh	60	Bursting	450 ± 112
RG Flx inh	140	Tonic	720 ± 180
RG Ext exc	80	Bursting	840 ± 210
RG Ext exc	720	Tonic	1344 ± 336
RG Ext inh	20	Bursting	840 ± 210
RG Ext inh	180	Tonic	1344 ± 336
$V1_{RG}$	300	Tonic	144 ± 36
V2b	300	Tonic	182 ± 45
V2a	158	Tonic	480 ± 120
V0c	15	Tonic	480 ± 120
Ia	60	Tonic	240 ± 60
RC	30	Bursting	0
Motoneuron	150	Tonic	0

Table A.2: Parameter values to produce tonic firing and bursting behavior. RG = Rhythm Generator, RC = Renshaw Cell

**Parameter**	**Tonic firing**	**Bursting (RG)**	**Bursting (RC)**
V_{th} (mV)	-50 ± 1	-51 ± 1	-51 ± 1
t_{ref} (ms)	9 ± 0.2	3 ± 0.2	3 ± 0.2
C (pF)	200 ± 40	600 ± 80	600 ± 80
g_L (nS)	10	26	26
E_L (mV)	-70	-60	-60
ΔT (mV)	2	3	2
τ_w (ms)	30	260	130
a (nS)	3	-11	-11
b (pA)	0	60	30
V_{reset} (mV)	-58	-48	-48

Appendix B. Statistics

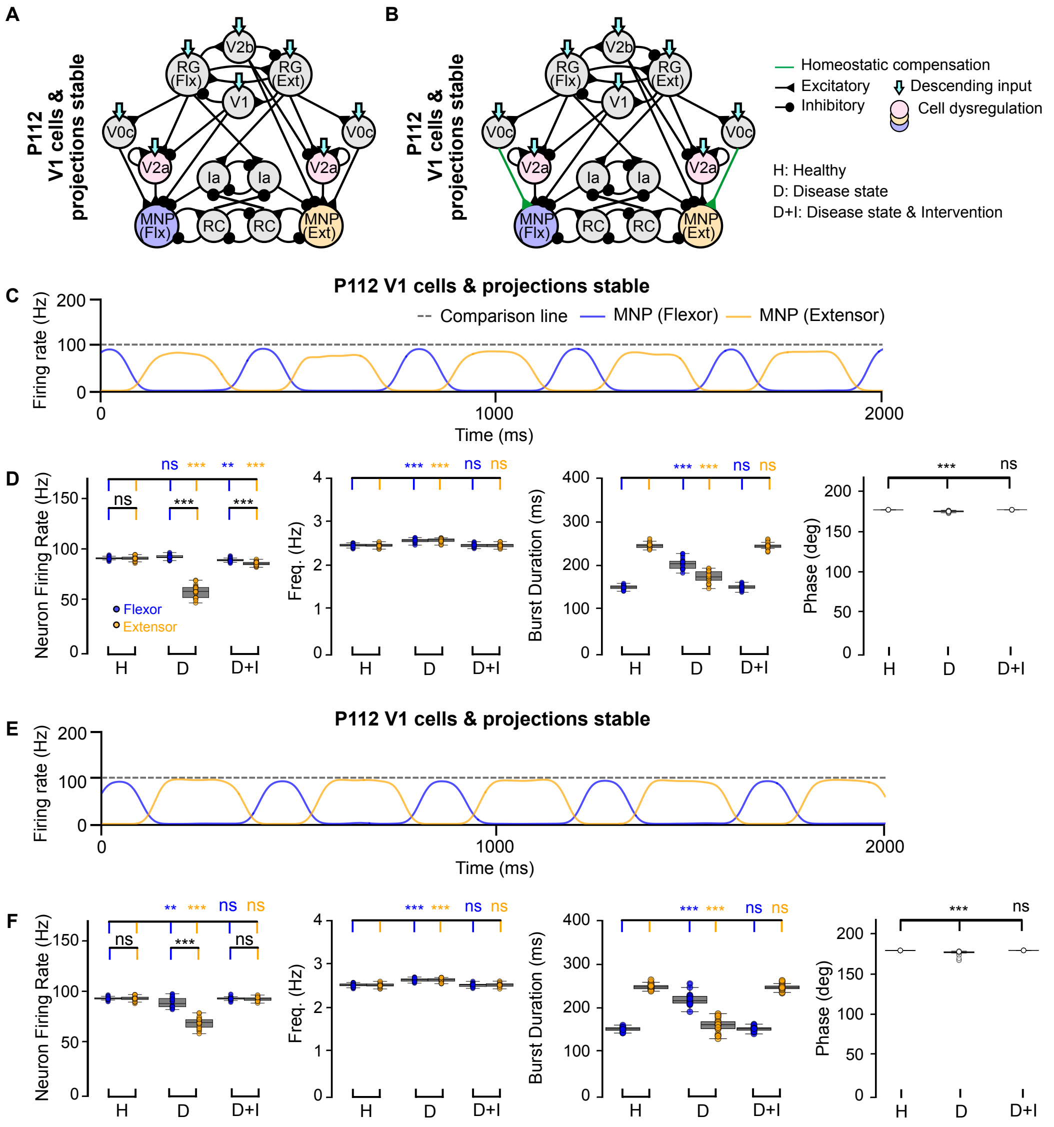

G

Mean and standard deviation per metric under test conditions

Timepoint	% Outlier	Max IFR Flx	Max IFR Ext	Freq Flx	Freq Ext	BD Flx	BD Ext	MNP Phase
**V1 cells and projections stable without V0c homeostatic compensation (comparing to healthy and no intervention)**								
P112	0	91.16±1.51	87.73±2.0	2.49±0.04	2.49±0.04	153.71±5.31	247.37±7.14	179.22±0.13
p-value vs healthy		1.93e-03	3.41e-04	1.00e00	1.00e00	1.00e00	1.00e00	9.42e-01
p-value flx vs ext		6.56e-06		3.97e-01		5.96e-08		
**V1 cells and projections stable with V0c homeostatic compensation (comparing to healthy and no intervention)**								
P112	0	92.89±1.40	92.36±1.84	2.49±0.04	2.49±0.04	153.76±5.09	247.71±6.78	179.29±0.12
p-value vs healthy		1.00e00	1.00e00	1.00e00	1.00e00	1.00e00	1.00e00	1.48e-01
p-value flx vs ext		2.52e-02		3.60e-01		1.19e-07		

Figure B.8: **Computational model of the locomotor CPG with V1 cells and projections stable but V2a interneuron and motoneuron loss. A,B**) Block diagram visualizing network changes. **C,E**) Example population output of the Flexor and Extensor motoneurons. **D,F**) Variance plots comparing neuron firing rate, frequency, burst duration, and phase. **G**) Statistics overview at P112 when V1 cells and projections are stable showing mean and standard deviation. P-values either compare the time point to healthy or flexor versus extensor within the time point. Significance across conditions was assessed with a Kruskal-Wallis H-test with Dunn's test post hoc, P-values comparing flexor versus extensor are calculated with a Wilcoxon Signed-Rank test, N=25, $p <= 0.001 =$ ***, $0.001 > p <= 0.01 =$ **, $0.01 > p <= 0.05$: *, $p > 0.05 =$ ns. The grey dotted line at 100Hz is used for comparison across all figures.

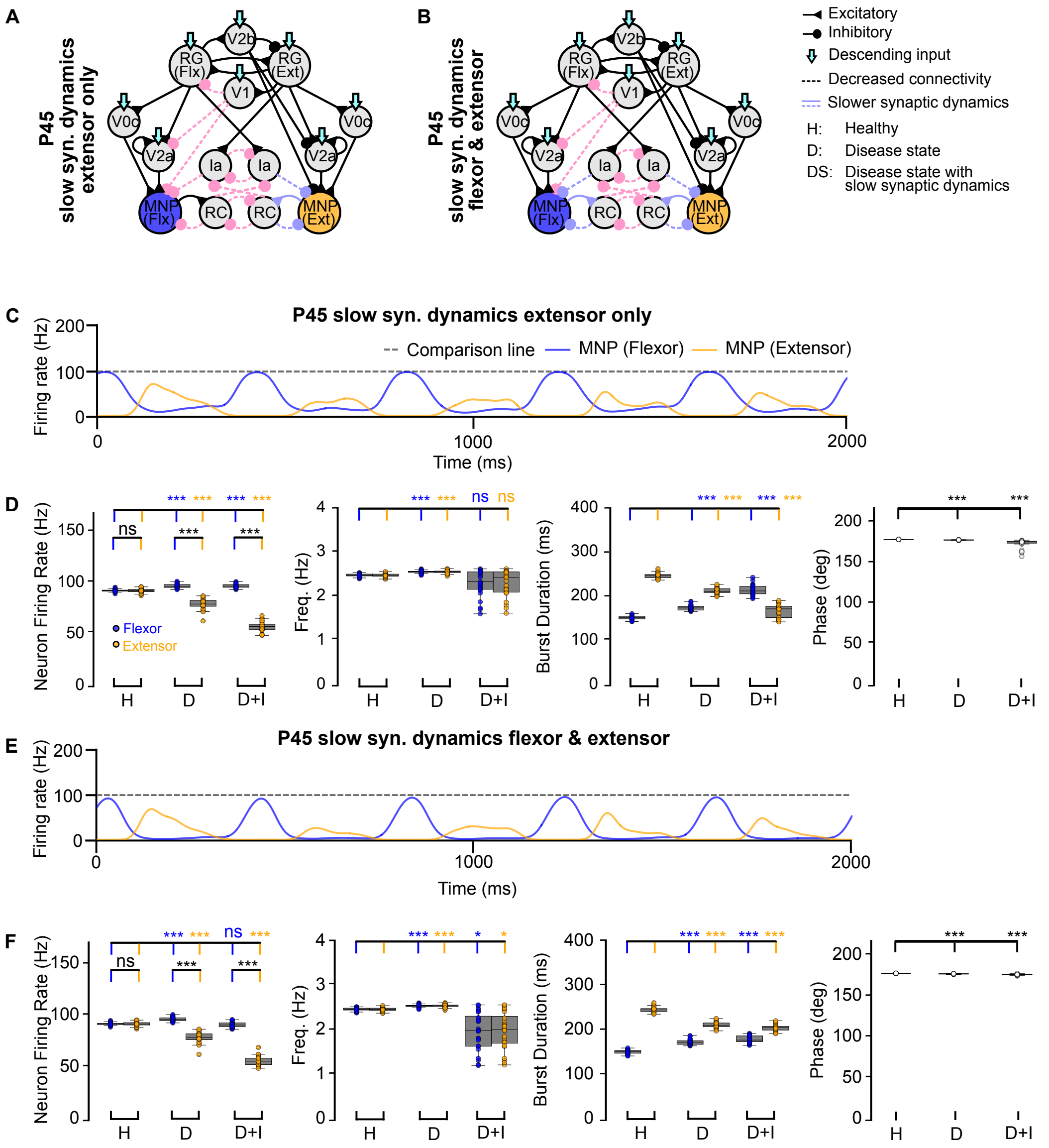

G

Mean and standard deviation per metric under test conditions

Timepoint	% Outlier	Max IFR Flx	Max IFR Ext	Freq Flx	Freq Ext	BD Flx	BD Ext	MNP Phase
**Increased rise and decay times to RCs and MNs - extensor side only (comparing to healthy and no intervention)**								
P45	0	97.48±1.95	58.2±4.88	2.31±0.3	2.31±0.03	215.5±11.63	169.38±14.9	173.73±5.5
p-value vs healthy		1.70e-08	3.10e-15	1.00e00	1.00e00	2.37e-15	2.37e-15	3.82e-15
p-value flx vs ext		5.96e-08		1.00e00		5.96e-08		
**Increased rise and decay times to RCs and MNs - flexor and extensor (comparing to healthy and no intervention)**								
P45	4	92.06±2.65	57.31±4.47	1.99±0.42	2.0±0.41	181.57±7.77	207.9±7.47	177.64±0.66
p-value vs healthy		1.00e00	4.21e-15	1.29e-02	2.38e-02	1.49e-11	1.23e-11	4.21e-13
p-value flx vs ext		1.19e-07		8.64e-01		1.19e-07		

Figure B.9: **Computational models of the locomotor CPG with slow synaptic dynamics. A,B**) Block diagram visualizing network changes. **C,E**) Example population output of the Flexor and Extensor motoneurons **D,F**) Variance plots comparing neuron firing rate, frequency, burst duration, and phase. **G**) Statistics overview at P45 during slow synaptic dynamics showing mean and standard deviation. P-values either compare the time point to healthy or flexor versus extensor within the time point. Significance annotation is calculated with a Kruskal-Wallis H-test with Dunn's test post hoc, P-values comparing flexor versus extensor are calculated with a Wilcoxon Signed-Rank test, N=25, $p <= 0.001$ = ***, $0.001 > p <= 0.01$ = **, $0.01 > p <= 0.05$: *, $p > 0.05$ = ns. The grey dotted line at 100Hz is used for comparison across all figures.

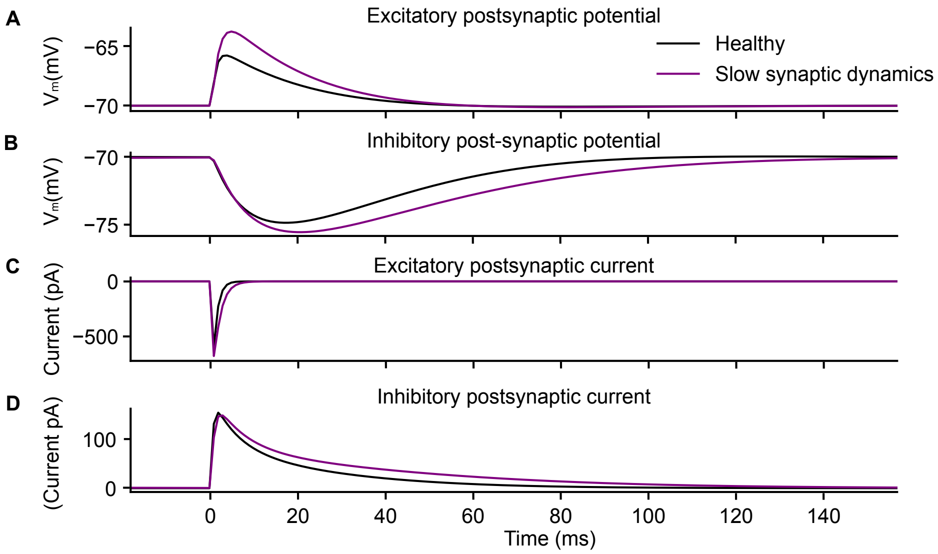

Figure B.10: **Comparison of healthy to slow synaptic dynamics.** Healthy (black) versus slow (purple) postsynaptic kinetics for excitatory and inhibitory synapses. **A**) Excitatory postsynaptic potential. **B**) Inhibitory postsynaptic potential. **C**) Excitatory postsynaptic current. **D**) Inhibitory postsynaptic current.

A

Mean and standard deviation per metric at progressive timepoints

Timepoint	% Outlier	Max IFR Flx	Max IFR Ext	Freq Flx	Freq Ext	BD Flx	BD Ext	MNP Phase
Healthy	0	92.91±1.25	92.93±2.09	2.49±0.03	2.49±0.04	153.29±4.49	248.35±6.14	179.18±0.12
p-value flx vs ext		9.16e-01		6.15e-01		5.96e-08		
**No intervention**								
P45	4	97.52±2.03	79.75±5.27	2.57±0.03	2.57±0.04	175.67±5.98	213.86±6.72	178.52±0.35
p-value flx vs ext		1.19e-07		8.86e-01		1.19e-07		
P63	8	95.85±2.16	82.93±4.00	2.59±0.03	2.59±0.03	263.72±35.35	122.15±24.99	165.49±10.27
p-value flx vs ext		2.38e-07		8.91e-01		2.38e-07		
P112	4	88.81±4.60	69.70±5.02	2.61±0.03	2.60±0.04	219.70±12.67	162.02±15.51	175.82±3.12
p-value flx vs ext		1.19e-07		5.43e-01		1.19e-07		

B

Mean and standard deviation per metric under test conditions

Timepoint	% Outlier	Max IFR Flx	Max IFR Ext	Freq Flx	Freq Ext	BD Flx	BD Ext	MNP Phase
**No intervention (comparing to healthy and no intervention)**								
MNs stable	4	90.47±4.1	71.35±6.02	2.61±0.04	2.61±0.03	216.55±9.21	167.20±11.17	177.26±0.70
p-value vs healthy		9.90e-02	6.67e-08	1.05e-08	1.88e-08	1.76e-08	4.66e-08	9.65e-08
V2a stable	4	101.58±1.78	91.62±2.37	2.61±0.04	2.61±0.04	373.05±167.53	85.32±23.17	143.33±22.93
p-value vs healthy		1.63e-06	5.61e-01	5.85e-09	5.04e-09	3.76e-15	3.76e-15	7.70e-15
V2a & MNs stable	8	102.27±1.54	91.98±2.75	2.60±0.03	2.61±0.03	318.91±73.27	91.03±25.57	150.29±22.27
p-value vs healthy		2.33e-06	1.00e00	2.97e-08	2.48e-08	6.91e-15	6.53e-15	3.04e-14

C

Mean and standard deviation per metric under test conditions

Timepoint	% Outlier	Max IFR Flx	Max IFR Ext	Freq Flx	Freq Ext	BD Flx	BD Ext	MNP Phase
**Exogenous synaptic stabilization (comparing to healthy and no intervention)**								
P45	4	89.92±1.80	88.30±3.84	2.49±0.03	2.49±0.03	151.06±4.93	250.08±5.16	179.19±0.16
p-value vs healthy		2.29e-03	2.34e-03	1.00e00	1.00e00	5.54e-01	1.00e00	1.00e00
P63	0	91.58±2.04	86.74±5.09	2.47±0.04	2.47±0.04	159.08±6.54	246.31±6.14	179.10±0.22
p-value vs healthy		2.19e-01	1.44e-04	3.20e-01	2.99e-01	1.32e-01	1.00e00	1.00e00
P112	12	89.94±2.33	84.58±2.65	2.48±0.04	2.48±0.04	169.63±9.10	233.71±11.14	179.27±0.15
p-value vs healthy		8.06e-04	2.97e-04	1.00e00	1.00e00	7.05e-04	7.67e-03	4.22e-01

D

Mean and standard deviation per metric under test conditions

Timepoint	% Outlier	Max IFR Flx	Max IFR Ext	Freq Flx	Freq Ext	BD Flx	BD Ext	MNP Phase
**Exogenous synaptic stabilization (comparing to healthy and no intervention)**								
MNs stable	0	90.86±2.37	85.15±3.9	2.48±0.05	2.48±0.05	168.55±11.51	234.95±11.82	179.17±0.23
p-value vs healthy		7.43e-03	1.85e-04	1.00e00	1.00e00	1.93e-03	1.74e-02	1.00e00
V2a stable	4	96.94±1.62	93.3±3.68	2.49±0.04	2.48±0.04	172.11±9.02	230.33±9.68	179.12±0.15
p-value vs healthy		1.58e-09	1.00e00	1.00e00	1.00e00	3.95e-04	1.04e-03	1.00e00
V2a & MNs stable	0	97.05±2.2	95.12±3.11	2.48±0.04	2.48±0.04	168.04±11.23	235.2±11.78	179.16±0.18
p-value vs healthy		1.78e-09	2.19e-01	1.00e00	1.00e00	4.43e-03	1.88e-02	1.00e00

Timepoint	Max IFR
MNs stable	
p-value flx vs ext	8.34e-07
V2a stable	
p-value flx vs ext	1.09e-03
V2a & MNs stable	
p-value flx vs ext	5.16e-02

E

Mean and standard deviation per metric under test conditions

Timepoint	% Outlier	Max IFR Flx	Max IFR Ext	Freq Flx	Freq Ext	BD Flx	BD Ext	MNP Phase
**Increased rise and decay time to RCs and MNs (comparing to healthy and no intervention)**								
P45	4	92.56±2.59	72.97±4.85	2.57±0.03	2.56±0.04	154.51±4.25	235.03±5.56	178.88±0.31
p-value vs healthy		1.00e00	5.25e-13	2.44e-07	2.42e-06	1.00e00	6.02e-04	8.20e-03
**Exogenous synaptic stabilization, Increased rise and decay times to RCs and MNs (comparing to healthy and no intervention)**								
P45	4	76.38±5.75	88.18±4.01	2.49±0.03	2.49±0.04	139.51±4.53	261.59±5.13	179.18±0.14
p-value vs healthy		1.14e-04	1.69e-03	1.00e00	1.00e00	4.14e-04	9.20e-04	1.00e00

Figure B.11: **Statistics per results panel.** **A**) Statistics overview at each time point without intervention showing mean and standard deviation. P-values compare flexor versus extensor within the time point. **B**) Statistics overview at P112 without and with synaptic stablization. P-values are in comparison to the healthy state. **C**) Statistics overview of each disease time point with synaptic stablization. P-values are in comparison to the healthy state. **D**) Statistics overview at P112 with synaptic stablization. P-values are in comparison to the healthy state as well as flexor versus extensor. **E**) Statistics overview at P45 with slow synaptic dynamics showing mean and standard deviation. P-values compare across conditions. P-values comparing across conditions are calculated with a Kruskal-Wallis H-test with Dunn's test post hoc, P-values comparing flexor versus extensor are calculated with a Wilcoxon Signed-Rank test, N=25.
